# MORPH-PRO: a novel algorithm and web server for protein morphing

**DOI:** 10.1186/1748-7188-8-19

**Published:** 2013-07-11

**Authors:** Natalie E Castellana, Andrey Lushnikov, Piotr Rotkiewicz, Natasha Sefcovic, Pavel A Pevzner, Adam Godzik, Kira Vyatkina

**Affiliations:** 1 Department of Computer Science, University of California-San Diego, La Jolla, CA USA; 2 Burnham Institute for Medical Research, North Torrey Pines Road, La Jolla, CA USA; 3 Joint Center for Structural Genomics, Bioinformatics Core, University of California-San Diego, La Jolla, CA USA; 4 Algorithmic Biology Laboratory, Saint Petersburg Academic University, Saint Petersburg, Russia

**Keywords:** Protein morphing, Molecular docking, Virtual screening

## Abstract

**Background:**

Proteins are known to be dynamic in nature, changing from one conformation to another while performing vital cellular tasks. It is important to understand these movements in order to better understand protein function. At the same time, experimental techniques provide us with only single snapshots of the whole ensemble of available conformations. Computational protein morphing provides a visualization of a protein structure transitioning from one conformation to another by producing a series of intermediate conformations.

**Results:**

We present a novel, efficient morphing algorithm, Morph-Pro based on linear interpolation. We also show that apart from visualization, morphing can be used to provide plausible intermediate structures. We test this by using the intermediate structures of a c-Jun N-terminal kinase (JNK1) conformational change in a virtual docking experiment. The structures are shown to dock with higher score to known JNK1-binding ligands than structures solved using X-Ray crystallography. This experiment demonstrates the potential applications of the intermediate structures in modeling or virtual screening efforts.

**Conclusions:**

Visualization of protein conformational changes is important for characterization of protein function. Furthermore, the intermediate structures produced by our algorithm are good approximations to true structures. We believe there is great potential for these computationally predicted structures in protein-ligand docking experiments and virtual screening. The Morph-Pro web server can be accessed at http://morph-pro.bioinf.spbau.ru.

## Background

The number of solved protein structures in PDB [1] has grown enormously in recent years. However, the function of many proteins is highly correlated with their movement. X-Ray crystallography, which contributes most of the structures in PDB, gives us only a static view of protein structure. Recent developments in computational protein morphing [2-4] provide visualization of a molecule transitioning from one conformation to another by producing a series of intermediate conformations. In this paper we present a novel, computationally efficient algorithm for generating intermediate structures between two solved conformations of the same protein. In addition, we explore the possibility that intermediate structures generated in the morphing procedure may also represent realistic approximations of the actual protein conformational change, including the structures of the intermediate conformations.

Various attempts to predict the trajectory of proteins through conformational space have been made. Some success has been achieved through the use of elastic network models [5,6]. However, the accuracy of these methods depends on the chosen starting conformation (either apo- or holo-) and collectivity of the atoms in the motion [7]. Other attempts require numerous iterations of energy-minimization [8], which can be computationally expensive. Molecular dynamics simulations [9] may also be useful in determining the nature of conformational changes, but currently require significant computing power. Furthermore, motion planning techniques can be adapted to model molecular motions [10-12], providing an attractive alternative to the mentioned approaches due to their efficiency.

The most widely-used application to produce protein morphs is the Morph Server developed by Krebs and Gerstein [8]. The goal of the Morph Server is to provide visualization and classification of protein movements. Our emphasis is on the fast generation of intermediate structures that represent realistic conformations.

Given two aligned proteins as input, our MORPH-PRO algorithm produces a series of intermediate conformations. We use linear interpolation, so that at each step every residue will move along the straight line between its current position and its ending position. Unfortunately, this can lead to biologically infeasible intermediate structures with atoms occupying the same space, incorrect bond lengths, and incorrect bond angles. Therefore, we use the atom positions generated by linear interpolation as a first approximation to the correct solution, and use a dynamic programming algorithm to ensure that certain biological constraints are satisfied. This produces structures which better resemble real proteins. Because these techniques are very efficient, our algorithm can produce many intermediate structures very quickly.

The intermediate structures produced by morphing algorithms show great promise in molecular docking [13]. Molecular docking, which uses computer simulations to model and score protein-ligand binding, is a critical tool for drug discovery. Protein flexibility is believed to play a significant role in ligand binding [14]. One method for including flexibility in the docking experiment is to perform ensemble docking [15], which uses multiple conformations of the protein for evaluation. Performing docking against several conformations of a protein has been shown to provide better screening results, than against a single static structure [16]. The intermediate structures produced by morphing algorithms may improve our ability to detect these ligands, and therefore aide in the development of drug-like molecules [17].

## Methods

In this section we analyze the simplest form of the morphing problem and present our MORPH-PRO algorithm. We designate *P*_*start*_ and *P*_*end*_ as the sequences of 3-D coordinates of the C *α* atoms for the starting and ending conformations. For simplicity, we assume that proteins *P*_*start*_ and *P*_*end*_ have an equal number of residues, and are aligned in 3-D. Later we will discuss the situation where *P*_*start*_ and *P*_*end*_ do not meet these conditions and will address various extensions to the simplest model of the protein morphing problem.

### Morphing algorithm

We represent a sequence of *n* points in 3-D (*n*-tuple) as a 3·*n* matrix (*p*_*ij*_), where *p*_*ij*_ is the *i*-th coordinate of the *j*-th point. Let *n* be the number of residues in *P*_*start*_ and *P*_*end*_. Given a parameter *α*, we define the *α*-intermediate of proteins *P* and *P*^′^ as (1−*α*)·*P*+*α*·*P*^′^. The simplest way to morph *P*_*start*_ into *P*_*end*_ is to generate intermediate reconstructions (1−*α*)·*P*_*start*_+*α*·*P*_*end*_ for 0<*α*<1. However, some *α*-intermediates may not look like real proteins, for example they may consist of consecutive C *α* atoms at biologically impossible distances. Below we show how to solve the protein morphing problem thereby transforming every intermediate reconstruction (being a sequence of *n* points) into a *protein-like* sequence of points. At each iteration, every point first moves by an appropriate distance towards its ending position, and then the obtained sequence of points is adjusted to become protein-like.

The pseudo code of the algorithm for generating *K* protein-like sequences *P*_1_ …,*P*_*K*_ of points is as follows:

Below we describe the algorithm for transforming a sequence of points *P* into a protein-like structure *Proteinize*(*P*).

### Optimal equidistant sequence problem

Given a sequence *P* of *n* points, we define *d*_*j*_(*P*) as the distance between the (*j*)-th and the (*j*+1)-th points in *P*: $d_{j}(P)=\sqrt{{\left(p_{1,j+1}-p_{1,j}\right)}^{2}+{\left(p_{2,j+1}-p_{2,j}\right)}^{2}+{\left(p_{3,j+1}-p_{3,j}\right)}^{2}}$. A sequence *P* is (*a*,*ϵ*)-equidistant if *a*−*ϵ* ≤ *d*_*j*_(*P*) ≤ *a*+*ϵ* for 1 ≤ *j* ≤ *n*−1. Protein structures exhibit a strict distance constraint between consecutive C *α* atoms that are 3.8 Å apart within an error margin of 0.1 Å. A sequence of points is *protein-like* if it is (3.8,0.1)-equidistant. We note that the consecutive C *α* atoms in cis-proline do not adhere to this distance rule, and these cases are not handled by our algorithm.

We define the distance *d*(*P*,*P*^′^) between two sequences *P* and *P*^′^, of *n* points each, as $\overset{n}{\underset{j=1}{\sum }}\overset{3}{\underset{i=1}{\sum }}{\left(p_{i,j}-p_{i,j}^{\prime }\right)}^{2}$. An (*a*,*ϵ*)-equidistant sequence *P*^′^ is called an *optimal (a*,*ϵ)-equidistant approximation* of *P* if *d*(*P*,*P*^′^) is minimum among all possible (*a*,*ϵ*)-equidistant sequences *P*^′^. Below we describe an approximate solution to the following problem:

**Optimal Equidistant Sequence Problem (OESP)**: Given a sequence of points, find its optimal equidistant approximation.

### Solving OESP

**Figure 1:**
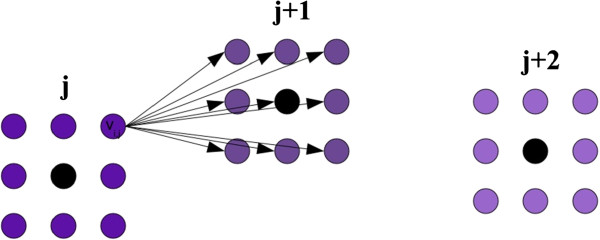
**Example lattices constructed around intermediate C*α* coordinates.** Lattices constructed in 2-D. The black vertices (*v*_0,*j*_, *v*_0,*j*+1_, *v*_0,*j*+2_) are the first approximations for the *j*^*th*^, (*j*+1)^*th*^, and (*j*+2)^*th*^ points. Each black vertex has a lattice constructed around it. Directed edges from a vertex *v*_*i*,*j*_ to all vertices in lattice (*j*+1) are also shown.

Here we describe an approximate OESP algorithm that assumes the space of possible solutions is discretized. For each point from the sequence *P*, we construct a lattice of 3-D points centered around it, as shown at Figure 1. Thus, each lattice is local to its corresponding point from *P*, which distinguishes our approach from naive and out-dated attempts to understand protein folding which utilize a global lattice [18-20]. The selection of the number of points in the lattice and the edge length is discussed later. Let *v*_*i*,*j*_ be the *i*^*th*^ vertex in the lattice constructed around the *j*^*th*^ point. Let *v*_0,*j*_ be the vertex corresponding to the *j*-th point in *P*. Let *Q* be the number of vertices in each lattice.

We construct a directed edge from a vertex *v*_*i*,*j*_ to a vertex *v*_*g*,*j*+1_ for 1 ≤ *i*,*g* ≤ *Q* and 1 ≤ *j* ≤ *n*−1. The score of an edge is defined as:

$$\;\text{EScore}(v_{i,j},v_{g,j+1})=\left\{\begin{array}{ll} 0, & \text{if}\;3.7\text{Å}\leq d(v_{i,j},v_{g,j+1})\leq 3.9\text{Å}\\ \infty , & \text{otherwise} \end{array}\right)$$

We also assign a score to each vertex, *v*_*i*,*j*_,

(1) $$\;\text{VScore}(v_{i,j})={\left(d(v_{i,j},v_{0,j})\right)}^{2}\;\text{for}\;1\leq i\leq Q\;\text{and}\;1\leq j\leq n,$$

where *d*(*v*_*i*,*j*_,*v*_0,*j*_) gives the distance between *v*_*i*,*j*_ and *v*_0,*j*_. Finding a protein-like sequence *P*^′^ of points which minimizes *d*(*P*,*P*^′^) translates into finding the path with the minimum score through the graph starting in the first lattice and ending in the *n*^*th*^ lattice. The score of a path is defined as the sum of the scores of its edges and vertices. Let *PATH*(*v*_*i*,*j*_) be the value of the minimum scoring path among those that start in the first lattice and end at vertex *v*_*i*,*j*_. Variable *PATH*(*v*_*i*,*j*_) can be computed using the following recurrence:

(2) $$\begin{array}{l} \text{PATH}(v_{i,1})=\text{VScore}(v_{i,1})\;\text{for}\;1\leq i\leq Q\\ \text{PATH}(v_{i,j})=\text{VScore}(v_{i,j})+\text{mi}n_{1\leq h\leq Q}\\ \;\;\;\{\text{PATH}(v_{h,j-1})+\text{EScore}(v_{h,j-1},v_{i,j})\} \end{array}$$

The score of the protein-like sequence of points which is closest to our original approximation is then

(3) $$\text{mi}n_{1\leq i\leq Q}\text{PATH}(v_{i,n})$$

The solution of OESP can be determined by backtracking. The time complexity of generating a protein-like conformation of C *α* atoms from a collection of *n* points, if one exists, is O(*nQ*^2^).

### Angle and proximity constraints

The above approach solves OESP and produces a (3.8,0.1)-equidistant sequence. There is more, however, to consider when defining a protein-like structure than consecutive residue distance. We now redefine the notion of a protein-like sequence of points to take into account consecutive residue angles and proximity constraints.

Given 3-D points *q*_1_, *q*_2_, and *q*_3_, a function *ang*(*q*_1_,*q*_2_,*q*_3_) is defined as the minor angle in degrees created by the lines through *q*_1_ and *q*_2_ and through *q*_2_ and *q*_3_, respectively. Given a sequence *P* of *n* points, we let *ang*_*j*_(*P*)=*ang*(*p*_*j*−1_,*p*_*j*_,*p*_*j*+1_) for 2 ≤ *j* ≤ *n*−1. A sequence *P* is (*a*,*b*)-angle consistent if *a*° ≤ *ang*_*j*_(*P*) ≤ *b*° for 2 ≤ *j* ≤ *n*−1. We observed that most C *α* angles in real proteins fall in the range of 70° to 120°.

Furthermore, a sequence *P* of points is *z*-distance consistent if the distance between any two non-consecutive points in *P* is at least *z* Å. We determined that a distance of 2.0 Å was typical in real proteins.

Finally, a sequence *P* is protein-like if it is (3.8,0.1)-equidistant, (70,120)-angle consistent, and 2.0-distance consistent.

We introduce a new score to evaluate the angle defined by three vertices, *v*_1_,*v*_2_, and *v*_3_.

$$\;\text{AScore}(v_{1},v_{2},v_{3})=\left\{\begin{array}{ll} 0, & \text{if}\;70\text{°}\leq \text{ang}(v_{1},v_{2},v_{3})\leq 120\text{°}\\ \infty , & \text{otherwise} \end{array}\right)$$

In order to incorporate angles into our algorithm, we must use a more complex recurrence which relies on both the current vertex, *v*_*i*,*j*_, and a preceding vertex, *v*_*h*,*j*−1_. We define *PATH*(*v*_*i*,*j*_,*v*_*h*,*j*−1_) as the path with minimum score among all paths that start in the first lattice, end in *v*_*i*,*j*_, and pass through *v*_*h*,*j*−1_. We replace (2) with the following for 1 ≤ *i*,*h* ≤ *Q*:

$$\begin{array}{l} \text{PATH}(v_{i,2},v_{h,1})=\text{VScore}(v_{i,2})+\text{EScore}(v_{h,1},v_{i,2})\\ \;\;+\text{VScore}(v_{h,1})\\ \text{PATH}(v_{i,j},v_{h,j-1})=\text{VScore}(v_{i,j})+\text{EScore}(v_{h,j-1},v_{i,j})\\ \;\;+\text{mi}n_{1\leq g\leq Q}\{\text{PATH}(v_{h,j-1},v_{g,j-2})\\ \;\;+\text{AScore}(v_{i,j},v_{h,j-1},v_{g,j-2})\} \end{array}$$

To determine the score of the protein-like sequence of points which is closest to our original approximation, we find:

$$\text{mi}n_{1\leq i,h\leq Q}\text{PATH}(v_{i,n},v_{h,n-1})$$

This construction does not force the sequence of points to be 2.0-distance consistent. For this, we apply a heuristic, which increases the *VScore* of vertices which are close to other lattices. We replace (1) with

$$\;\text{VScore}(v_{i,j})=\left\{\begin{array}{ll} {\left(d(v_{i,j},v_{0,j})\right)}^{2},\;\text{if}\;d(v_{i,j},v_{0,j})>2.0\\ {\left(d(v_{i,j},v_{0,j})\right)}^{2}+100\overset{j-2}{\underset{m=1}{\sum }}{\left(d(v_{i,j},v_{0,m})\right)}^{-2}, & \text{otherwise} \end{array}\right)$$

We chose the multiplier 100 because it worked well to prevent C *α* clashes in our morphs. The addition of the angle and distance constraints requires O(*n*^2^*Q*^3^).

However, the advanced strategy described above may be impractical if the proteins being examined are large or the conformational change is dramatic. Therefore, we also considered a simplified strategy which can significantly improve the running time. In the simplified strategy, (2) is replaced with

(4) $$\begin{array}{ll} \text{PATH}(v_{i,j})=\text{VScore}(v_{i,j})+\text{mi}n_{1\leq h\leq Q}\\ \;\{\text{PATH}(v_{h,j-1})+\\ \;\text{EScore}(v_{h,j-1},v_{i,j})+ & \\ \;\text{AScore}(\text{pre}v_{\text{PATH}}(v_{h,j-1}),v_{h,j-1},v_{i,j})\}, \end{array}$$

where *prev*_*PATH*_(*v*_*h*,*j*−1_) is the vertex preceding *v*_*h*,*j*−1_ in the best path ending at *v*_*h*,*j*−1_, the score of which is determined by the value of *PATH*(*v*_*h*,*j*−1_). Similar to the the basic method, the score of the optimal protein-like sequence of points is

(5) $$\text{mi}n_{1\leq i\leq Q}\text{PATH}(v_{i,n}),$$

and thus, the time complexity of the simplified strategy is also *O*(*nQ*^2^).

The simplified strategy may provide a sub-optimal intermediate structure. However, if a structure is produced, it obeys both the angle and proximity constraints. It should be noted that the simplified strategy may fail to find a solution to OESP instances, even when a solution can be found by the advanced algorithm. The advanced algorithm looks for an optimal path among *all* feasible ones stretching from the first to the last lattice, while the former takes into consideration only a subset of paths. In addition, the simplified strategy may require an increase of the lattice size (see Parameter Selection), thus reducing the difference in the running time in practice of the algorithms.

Our experiments described in detail below were carried out using the simplified strategy.

### Preprocessing

Our algorithm only interpolates intermediate positions for residues which are aligned. Therefore, if the input proteins have different lengths we use the Needleman-Wunsch global sequence alignment algorithm [21] to align them, and reduce our starting and ending conformations to include only positions that are aligned. We chose to use a sequence-based alignment method because *P*_*start*_ and *P*_*end*_ are likely related proteins and will have similar sequences. The output of this phase of the algorithm is a set of coordinates of aligned C *α*’s for *P*_*start*_ and *P*_*end*_. In this situation, the *i*^*th*^ residue in the alignment may not correspond to the *i*^*th*^ residue in *P*_*start*_. If the *i*^*th*^ and (*i*+1)^*st*^ residues produced from the alignment are not consecutive in *P*_*start*_ then *EScore* for the edge connecting them is 0. Similarly, if either the (*i*−1)^*th*^ and *i*^*th*^ or the *i*^*th*^ and (*i*+1)^*st*^ residues are not consecutive in *P*_*start*_ then *AScore* for the angle at the *i*^*th*^ residue is 0.

In order for the morphing algorithm to work, the proteins should be aligned in 3-D using a structure alignment program. In the implementation we used for the experiments described in this paper, this task is accomplished by Kabsch’s algorithm [22] (also see [23]). Our server uses the Quaternion Characteristic Polynomial (QCP) method recently proposed by [24].

### Parameter selection

For our experiments we set the number of intermediate structures, *K*, to be the rounded displacement of the largest C *α* movement. For example, if the greatest movement of any C *α* from the starting conformation and the ending conformation is 15.2 Å, then *K* = 15. This results in only small differences between consecutive structures.

We selected the edge length and point density for the lattices based on experimental evidence. Increasing the density of vertices in the lattice allows for a finer grained set of possible coordinates, but we found that a density higher than 6 points per Å (216 points per Å^3^) does not produce significantly better intermediate structures. Consequently, we fixed the density at 6 points per Å. The length of the lattice edge is set initially to 1 Å. However, if OESP solution cannot be found at this lattice size, we increase the lattice edge length (to 1.5 Å and then to 2.0 Å). If an OESP solution cannot be found with lattice edge length of 2.0 Å then our algorithm will not produce a morph.

### Server implementation

We implemented the MORPH-PRO server using an open source web framework Ruby on Rails and SQLite3 database engine, and a new 3D graphics standard WebGL. The algorithm for protein morphing was implemented in ANSI C. We used BioRuby [25] – an open source bioinformatics library for Ruby – for parsing PDB files, and the QCProt 1.3 realization of the QCP algorithm for aligning proteins in 3D, distributed under a BSD open source license.

The interface allows a user to upload two PDB files containing the starting and the ending conformations, and either to explicitly indicate the number of intermediate conformations or to let it be determined automatically (based on the maximum C *α* displacement, as described in Parameter Selection). After the intermediate conformations are computed, the morphing process can be visualized either as a movie or step-by-step. A transformation between two consecutive conformations is accomplished via linear interpolation. A 3D chain representing a conformation can be rotated, and zoomed in and out. In addition, a user can choose an appropriate level of detail for rendering and elect to use the full algorithm or the simplified version. A publicly available archive of submitted morph requests is stored on the server in an SQLite3 database, making it easier to re-run the algorithm on the same input.

## Results and discussion

We evaluate our morphs by looking at both the biological feasibility of each individual structure, as well as the series of structures as a whole. We evaluate our morphs by comparing to proteins which have 3 or more solved structures in PDB, as proposed by [26]. In many instances, multiple conformations of the same protein are not available. Instead, we used proteins from the same family with nearly identical sequences as endpoints in our morph.

### Pyrophosphokinases

We created a morph between two members of the pyrophosphokinase family (PDB codes: 1DY3, 1RAO). The alignment produced 158 residues with a maximum C *α* displacement of 22 Å. The RMSD between the starting structure and the ending structure is 4.07 Å.

**Figure 2:**
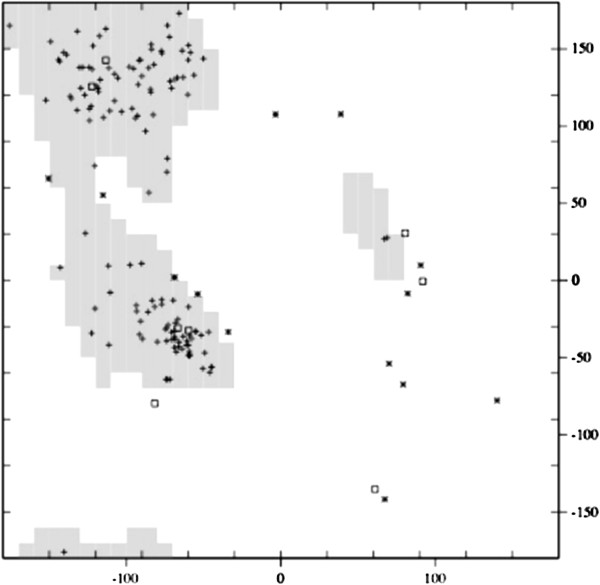
**Ramachandran plot of intermediate structures for pyrophosphokinase morph.** The Ramachandran plot [27] of the intermediate structure which occurs halfway in the morph from 1DY3 to 1RAO. Angles that occur in the core regions are represented as plus signs while outliers are represented as asterisks. Glycines are represented as squares. The absent atoms in the backbone and side chains of each intermediate structure were reconstructed using Maxsprout [28], and energy minimization was performed using Swiss-PDB viewer [29].

We examined each intermediate structure produced from this morph, and looked for clashing C *α* atoms. None of the intermediate structures had atoms within 2 Å of another atom. We also looked at torsion angles created by C *α* atoms. The Ramachandran plot of phi versus psi angles of the intermediate structure, which occurs halfway through the morph, is shown in Figure 2. The majority of the points in the plot fall within a region that is observed in real proteins. This indicates that our structure exhibits characteristics of real proteins.

It is also beneficial to look at the intermediate structures in the context of the entire morph. We have shown that our intermediates are protein-like, and we now demonstrate that the series of intermediate structures closely mimics the series of conformations a protein would visit. If multiple conformations of the same protein are known, then we can compare our predicted trajectory to the solved trajectory by calculating the RMSD between our intermediates and the experimentally solved intermediates. However, alternate conformations were not available for these proteins, so instead we used solved structures for proteins in the pyrophosphokinase family.

**Figure 3:**
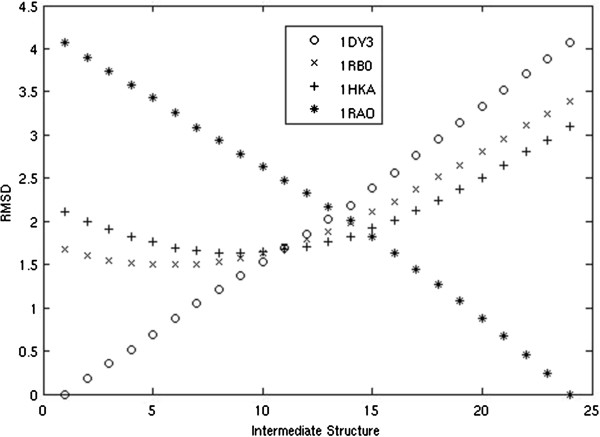
**RMSD of 22 intermediate structures to solved pyrophosphokinase structures.** RMSD of 22 intermediate structures, the starting protein, and the ending protein to 1DY3, 1RB0, 1HKA, and 1RAO.

We chose two additional pyrophosphokinases to act as ‘experimental’ intermediates (PDB codes: 1RB0, 1HKA). We chose these proteins because they can be ordered by their RMSD between 1DY3 and 1RAO, and therefore are likely to be similar to the trajectory the morph should take. We plot the RMSD of our intermediate structures against each of these four proteins in Figure 3.

Intermediates which are produced early in the morph are closest to the starting protein, 1DY3, while those that are produced late in the morph are closest to the ending protein, 1RAO, as expected. Our intermediates from the middle of the morph become close to both ‘experimental’ intermediates, 1RB0 and 1RAO, suggesting that our movement closely follows the evolutionary changes which occurred between the two proteins. In addition, the intermediate structures generated by our algorithm come roughly as close, if not closer, to the known homologs as those produced by Morph Server, as demonstrated in Table 1. A direct speed test with the Morph Server was not possible because a fully functional standalone tool was not available.

**Table 1:** RMSD of predicted structures to solved intermediate structures

***Intermediate***	***RMSD to 1RB0(Å)***		***RMSD to 1HKA(Å)***	
***Structure***	***Morph***	***Morph server***	***Morph***	***Morph server***
1	1.679	1.679	2.108	2.091
2	1.548	1.531	1.903	1.878
3	1.501	1.458	1.759	1.726
4	1.509	1.517	1.655	1.683
5	1.639	1.668	1.643	1.717
6	1.886	1.903	1.760	1.840
7	2.105	2.218	1.919	2.064
8	2.511	2.604	2.246	2.392
9	2.957	2.986	2.655	2.745
10	3.390	3.390	3.101	3.127

The RMSD of 10 intermediate structures produced by MORPH-PRO and the Morph Server to the experimental’ intermediates of the starting and ending conformations.

### F1-ATPase

The technique of looking at RMSD of the intermediate structures to known structures is most useful when X-Ray structures of actual intermediate conformations are available. There are three conformations solved for the F1-ATPase molecular motor (PDB code: 1E79) which exhibit a subtle change. The RMSD between the starting and ending conformations is 1.78 Å. The protein has 492 residues and the largest movement of a C *α* is 11 Å. We produce a morph of 11 total structures from 1E79A to 1E79C.

**Figure 4:**
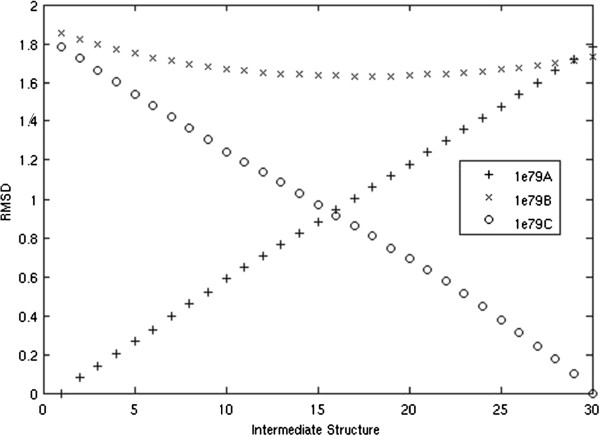
**RMSD of 30 intermediate structures to solved intermediate structures of F1-ATPase molecular motor.** RMSD of 30 intermediate structures to 1E79A, 1E79B, and 1E79C.

The intermediate structures are very similar to all of the known structures, with RMSD consistently less than 2 Å. We do, however, see our intermediate structures become closer to the known intermediate 1E79B. One intermediate structure comes as close as 1.61 Å, while the starting structure (1E79A) is 1.85 Å and the ending structure (1E79C) is 1.73 Å. Figure 4 demonstrates how the predicted intermediates are similar to the starting structure early in the morph, become more similar to the known intermediate structure in the middle of the moprh, and then finally become similar to the ending structure. In Figure 4, we generated 30 intermediate structures to better illustrate this point.

### GroEL

**Figure 5:**
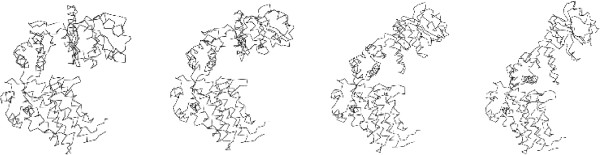
**The visualization of the morph predicted for GroEL.** The initial conformation, 2 intermediate structures, and the final conformation for GroEL.

Our algorithm also performs well on large proteins. GroEL proteins chaperon the folding of other proteins. Two GroEL proteins (PDB codes: 1GRL and 1AON) exhibit a simple morph on 515 aligned residues, changing from a closed conformation to an open conformation. The RMSD between these two structures is 12.36 Å while the largest movement of a single C *α* is 34.8 Å. Despite the large number of atoms and the significant movement, the morph took only a couple minutes to run. Figure 5 shows the initial conformation, the final conformation and 2 out of 34 intermediate structures produced in this morph.

### Virtual screening

Virtual screening [30] is a technique which simulates the binding of a protein and a ligand, in order to determine the best ligand candidates from a large database. Most often, virtual screening is used as part of a drug development pipeline, guiding the selection of likely drug candidates. The predicted binding affinity of a ligand for a protein is determined by a docking algorithm, which finds the orientation and location of the ligand with respect to the protein. Modeling protein flexibility is very difficult due to the large degrees of freedom of a protein structure [13,31]. One promising approach to implicitly incorporating protein flexibility is to dock against an ensemble of static protein structures [32].

If multiple conformations of the target protein are solved using NMR or X-Ray studies, these are good candidates for ensemble docking. However, in the more common case of unknown intermediate conformations a computational method can provide accurate models more quickly. Use of computationally-produced intermediates in virtual screening has shown promising results [33].

To test the potential for our intermediate structures in virtual screening we examined docking scores of our structures versus those solved experimentally against a small database of ligands. First, we produced a morph of the c-Jun N-terminal kinase 1 (JNK1). The starting conformation of this protein (1UKH) was solved complexed with a peptide (pepJIP1) derived from the binding portion of the scaffolding protein JIP1. The ending conformation (1UKI) was solved complexed with pepJIP1 and the ATP mimic SP600125. The binding of pepJIP1 to the JIP1 binding site on JNK1 causes a small conformational change at the ATP site. Though the movement is small, it produces a morph of 3 intermediates (*P*_2_,*P*_3_,*P*_4_) in addition to the starting and ending conformations. The absent backbone atoms and side chains of each intermediate structure were reconstructed using Maxsprout [28], and energy minimization was performed using Swiss-PDB viewer [29]. As a basis for comparison, the X-Ray structures of 1UKH and 1UKI were also reduced to their C *α*’s and then reconstructed in the same manner to produce *P*_1_ and *P*_5_, respectively.

Next, we performed docking with GOLD [34], a commonly used docking program and scoring scheme, on four ligands (extracted from PDB) known to bind to JNK1, as well as SP600125. Table 2 shows the rankings of the binding affinities from highest to lowest based on the GoldScore. The headings are the PDB codes for the solved structures of JNK1 complexed with each ligand.

**Table 2:** Binding affinities for 5 JNK1 putative ligands

***SP600125***	***2G01***	***2N03***	***2H96***	***2GMX***
1*UKI*	*P*_5_	*P*_2_	*P*_2_	*P*_2_
1*UKH*	*P*_2_	*P*_5_	*P*_5_	*P*_5_
*P*_2_	*P*_4_	1*UKI*	1*UKI*	1*UKH*
*P*_5_	1*UKI*	*P*_3_	*P*_3_	1*UKI*
*P*_3_	*P*_1_	1*UKH*	1*UKH*	*P*_3_
*P*_4_	1*UKH*	*P*_4_	*P*_4_	*P*_1_
*P*_1_	*P*_3_	*P*_1_	*P*_1_	*P*_4_

The rankings of binding affinities for 5 ligands against the predicted intermediate structures and the two solved structures for JNK1.

The first column behaves as expected. The structure which has the highest binding affinity for SP600125 is 1UKI which is the structure of JNK1 complexed with SP600125. The X-Ray structures docked with SP600125 rank significantly higher than the reconstructed *P*_1_ and *P*_5_. This suggests that better side chain reconstruction could greatly improve the docking results.

For three of the other ligands, the second intermediate structure, *P*_2_ scores higher than any other intermediate structure as well as any X-Ray structure. This demonstrates that our intermediate structures would be more likely to identify ligands which bind to JNK1 than either of the two X-Ray structures.

## Conclusions

It is clear that there is much to learn about the nature of protein structure dynamics that is not addressed in the static information contained in PDB. The intermediate structures representing a protein as it moves from one conformation to another may yield much information about how a protein functions. Experimental techniques are inadequate for this task due to practical and technological limitations. For this reason, structural biology is in great need of algorithms which can accurately predict the intermediate structures as a protein undergoes a conformational change.

While other morphing algorithms require computationally expensive energy and elastic network modeling calculations, our morphing algorithm is based on a few simple observations of protein structure, and therefore produces multiple intermediate conformations very quickly. Our intermediate structures represent possible protein structures, and demonstrate the motion of a protein as it changes between conformations. In the case of morphing between homologs, the intermediate structures give us clues to how protein structures have evolved.

The morphed structures also show promise in the area of virtual screening. Most techniques limit protein flexibility to the side chain atoms, and may allow limited flexibility of the substrate. Our morph produces intermediate structures which are hypotheses for possible backbone movements. For this reason, some ligands bound more favorably to our intermediate structures than the solved structures. These are strong implications for the potential of morphs in guiding drug development.

Like all other approaches, our algorithm also has limitations. Linear interpolation, with only small corrections, prevents our method from correctly producing a morph for proteins with very large or complex movements. Many of these morphs could be solved by allowing a larger movement from the first approximation (a larger lattice), or allowing higher granularity of possible C *α* positions (more points in each lattice) but the time cost would be significant. Clearly, in protein morphing there is a trade-off between speed and accuracy.

## Abbreviations

RMSD: Root mean square deviation; PDB: Protein Data Bank.

## Competing interests

The authors declare no conflicts of interests.

## Authors’ contributions

The algorithm was developed by NEC, PAP, PR, NS, and AG. The experiments were performed by NEC. The Morph-Pro server was developed by AL and KV. The manuscript was prepared by NEC. PAP and KV contributed to finalizing the manuscript. All authors read and approved the final manuscript.

## References

[B1] Berman HM Westbrook J Feng Z Gilliland G Bhat TN Weissig H Shindyalov IN Bourne PE: The protein data bank Nucleic Acids Res 2000 28 235 242 10.1093/nar/28.1.235 10592235 PMC102472

[B2] Echols N Milburn D Gerstein M MolMovDB: analysis and visualization of conformational change and structural flexibility Nucleic Acids Res 2003 31 478 482 10.1093/nar/gkg104 12520056 PMC165551

[B3] Kim MK Jernigan RL Chirikjian GS Efficient generation of feasible pathways for protein conformational transitions Biophys J 2002 83 1620 1630 10.1016/S0006-3495(02)73931-3 12202386 PMC1302259

[B4] Kim MK Chirikjian GS Jernigan RL Elastic models of conformational transitions in macromolecules J Mol Graph Model 2002 21 151 160 10.1016/S1093-3263(02)00143-2 12398345

[B5] Franklin J Koehl P Doniach S Delarue M MinActionPath: maximum likelihood trajectory for large-scale structural transitions in a coarse-grained locally harmonic energy landscape Nucleic Acids Res 2007 35 Web Server issue W477 W482 17545201 10.1093/nar/gkm342 PMC1933200

[B6] Ahmed A Gohlke H Multiscale modeling of macromolecular conformational changes combining concepts from rigidity and elastic network theory Proteins 2006 63 1038 1051 10.1002/prot.20907 16493629

[B7] Yang L Song G Jernigan RL How well can we understand large-scale protein motions using normal modes of elastic network models? Biophys J 2007 93 920 929 10.1529/biophysj.106.095927 17483178 PMC1913142

[B8] Krebs WG Gerstein M The morph server: a standardized system for analyzing and visualizing macromolecular motions in a database framework Nucleic Acids Res 2000 28 1665 1675 10.1093/nar/28.8.1665 10734184 PMC102811

[B9] Duan Y Kollman PA Pathways to a protein folding intermediate observed in a 1-Microsecond simulation in aqueous solution Science 1998 282 5389 740 744 9784131 10.1126/science.282.5389.740

[B10] Amato NM Song G Using motion planning to study protein folding pathways J Comput Biol 2002 9 2 149 168 10.1089/10665270252935395 12015875

[B11] Apaydin MS Brutlag DL Guestrin C Hsu D Latombe JC Varma C Stochastic roadmap simulation: an efficient representation and algorithm for analyzing molecular motion J Comput Biol 2003 10 3–4 257 281 12935328 10.1089/10665270360688011

[B12] Raveh B Enosh A Schueler-Furma O Halperin D Rapid sampling of molecular motions with prior information constraints PLoS Comput Biol 2009 5 2 e1000295 10.1371/journal.pcbi.1000295 19247429 PMC2637990

[B13] Teodoro ML Kavraki LE Conformational flexibility models for the receptor in structure based drug design Curr Pharm Des 2003 9 1635 1648 10.2174/1381612033454595 12871062

[B14] Carlson HA Protein flexibility and drug design: how to hit a moving target Curr Opin Chem Biol 2002 6 447 452 10.1016/S1367-5931(02)00341-1 12133719

[B15] Knegtel RM Kuntz ID Oshiro CM Molecular docking to ensembles of protein structures J Mol Biol 1997 266 424 440 10.1006/jmbi.1996.0776 9047373

[B16] Craig IR Essex JW Spiegel K Ensemble docking into multiple crystallographically derived protein structures: an evaluation based on the statistical analysis of enrichments J Chem Inf Model 2010 50 511 524 10.1021/ci900407c 20222690

[B17] Goh CS Milburn D Gerstein M Conformational changes associated with protein-protein interactions Curr Opin Struct Biol 2004 14 104 109 10.1016/j.sbi.2004.01.005 15102456

[B18] Taketomi H Ueda Y Go N Studies on protein folding, unfolding and fluctuations by computer simulation Int J Peptide Protein Res 1975 7 6 445 459 1201909

[B19] Lau KF Dill KA A lattice statistical mechanics model of the conformational and sequence spaces of proteins Macromolecules 1989 22 10 3986 3997 10.1021/ma00200a030

[B20] Sali A Shakhnovich E Karplus M How does a protein fold? Nature 1994 369 248 251 10.1038/369248a0 7710478

[B21] Needleman SB Wunsch CD Needleman-Wunsch algorithm for sequence similarity searches J Mol Biol 1970 48 443 453 10.1016/0022-2836(70)90057-4 5420325

[B22] Kabsch W A solution for the best rotation to relate two sets of vectors Acta Crystallogr Section A 1976 32 6 922 923

[B23] Ye Y Godzik A Flexible structure alignment by chaining aligned fragment pairs allowing twists Bioinformatics 2003 19 suppl 2 ii246 ii255 10.1093/bioinformatics/btg1086 14534198

[B24] Liu P Agrafiotis DK Theobald DL Fast determination of the optimal rotational matrix for macromolecular superpositions J Comput Chem 2010 31 7 1561 1563 20017124 10.1002/jcc.21439 PMC2958452

[B25] Goto N Prins P Nakao M Bonnal R Aerts J Katayama T BioRuby:bioinformatics software for the Ruby programming language Bioinformatics 2010 26 2617 2619 10.1093/bioinformatics/btq475 20739307 PMC2951089

[B26] Weiss DR Levitt M Can morphing methods predict intermediate structures? J Mol Biol 2009 385 665 674 10.1016/j.jmb.2008.10.064 18996395 PMC2691871

[B27] Kleywegt GJ Jones TA Phi/psi-chology: Ramachandran revisited Structure 1996 4 12 1395 1400 10.1016/S0969-2126(96)00147-5 8994966

[B28] Holm L Sander C Database algorithm for generating protein backbone and side-chain co-ordinates from a C alpha trace application to model building and detection of co-ordinate errors J Mol Biol 1991 218 183 194 10.1016/0022-2836(91)90883-8 2002501

[B29] Guex N Peitsch MC SWISS-MODEL and the Swiss-PdbViewer: an environment for comparative protein modeling Electrophoresis 1997 18 15 2714 2723 10.1002/elps.1150181505 9504803

[B30] Walters WP Stahl MT Murco MA ChemInform abstract: virtual screening-an overview ChemInform 1998 29 38 160 178

[B31] Teague SJ Implications of protein flexibility for drug discovery Nat Rev Drug Discov 2003 2 527 541 10.1038/nrd1129 12838268

[B32] Wei BQ Weaver LH Ferrari AM Matthews BW Shoichet BK Testing a flexible-receptor docking algorithm in a model binding site J Mol Biol 2004 337 1161 1182 10.1016/j.jmb.2004.02.015 15046985

[B33] Broughton HB A method for including protein flexibility in protein-ligand docking: improving tools for database mining and virtual screening J Mol Graph Model 2000 18 247 257 10.1016/S1093-3263(00)00036-X 11021541

[B34] Jones G Willett P Glen RC Leach AR Taylor R Development and validation of a genetic algorithm for flexible docking J Mol Biol 1997 267 3 727 748 10.1006/jmbi.1996.0897 9126849

